# Purification and characterization of thrombin from camel plasma: interaction with camel tick salivary gland thrombin inhibitor

**DOI:** 10.1186/s43141-023-00464-2

**Published:** 2023-01-23

**Authors:** Mahmoud A. Ibrahim, Hassan M. M. Masoud

**Affiliations:** 1 Molecular Biology Department, National Research Centre, Dokki, Giza, Egypt; 2 Proteome Research Laboratory, Central Laboratories Network and Centers of Excellence, National Research Centre, El-Tahrir St, Dokki, Giza, Egypt

**Keywords:** Thrombin, Camel plasma, Purification and characterization, Camel tick, Salivary gland, Thrombin inhibitor

## Abstract

**Background:**

Thrombin is the most important enzyme in the hemostatic process by permitting rapid and localized coagulation in case of tissue damage. Camel thrombin is the natural and proper target enzyme for the previously purified camel tick salivary gland thrombin inhibitor.

**Results:**

In this study, the camel thrombin was purified homogenously in a single affinity chromatographic step on the heparin-agarose affinity column with a specific activity of 3242 NIH units/mg proteins. On SDS-PAGE, the purified camel thrombin contained two forms, 37 kDa *α*-thrombin and 28 kDa *β*-thrombin, and the camel prothrombin was visualized as 72 kDa. The camel thrombin *Km* value was found out as 60 µM of N-(*p*-Tosyl)-Gly-Pro-Arg-*p*-nitroanilide acetate and displayed its optimum activity at pH 8.3. The PMSF was the most potent inhibitor of camel thrombin. Camel tick salivary gland thrombin inhibitor has two binding sites on camel thrombin and inhibited it competitively with Ki value of 0.45 µM.

**Conclusions:**

The purified camel thrombin was found to be more susceptible toward the camel tick salivary gland thrombin inhibitor than bovine thrombin.

## Background

Blood coagulation is a basic physiological defense mechanism that occurs in all vertebrates to prevent blood loss following vascular injury, and hemostasis is a tightly regulated mechanism that ensures the maintenance of blood flow under physiological conditions. A delicate balance exists between four major components to keep the fluid nature of blood: vascular endothelium, platelets, the coagulation pathway, and fibrinolysis [1–5]. All vertebrates have fine control of their hemostatic system, where any disturbance can cause thrombosis or bleeding events [6–8]. In all species, the basic mechanism of clot formation is similar when the endothelium is damaged; a complex sequence of enzymatic reactions occurs that is localized to the site of trauma and involves both activated cells and plasma proteins. Initiation of the reaction sequence is achieved by an expression of a tissue factor on the surface of activated cells that leads to thrombin generation, the most significant enzyme in the process of coagulation. Thrombin is a serine protease of two polypeptides, *α*- and *β*-chains, which is centrally implicated in the final step of blood coagulation process [9–12]. Thrombin converts fibrinogen to fibrin forming the blood clot matrix and exerting a positive feedback regulation for effective promotion of additional thrombin generation to facilitate a rapid thrombus formation [13–16]. Thrombin is a very important enzyme for the hemostatic process and is produced on request from its circulating zymogen prothrombin in response to different hemostatic activators. The successive and coordinated interactions of coagulation proteins which induce and magnify thrombin formation are controlled by a group of circulating anticoagulants or inhibitory proteins that function to ensuring thrombin generation is limited to areas of vascular injuries, and no excess thrombin is generated [17, 18].

Thrombin is one of the most powerful activators which trigger platelet aggregation [19],therefore, the large plurality of anticoagulants aims thrombin or factor Xa in the blood coagulation cascade [20–23]. Anticoagulants are considered to be fundamental for the succeeded feeding of blood-sucking animals via inhibition of clot formation at feeding sites in mouthparts and gut and by indirect inhibition of platelet aggregation via inhibiting thrombin generation [24–27]. Thrombin has high substrate specificity through its active site selectivity and via exosite I, a strong positively charged area on its surface, which is involved in thrombin-substrate interactions. Tick molecules inhibit thrombin by targeting exosite I using different mechanisms to prevent its binding to its natural substrates [22, 28]. Therefore, the present study aims at purification and characterization of the camel blood coagulation factor thrombin and the evaluation of the susceptibility of the camel thrombin as the natural and proper target enzyme with the previously purified camel tick salivary gland thrombin inhibitor [20].

## Methods

### Preparation of the camel plasma

For obtaining the plasma, a mixture of camel blood (900 ml) and 0.11 M sodium citrate solution (100 ml) was centrifuged for 15 min (2700 × g and 4 °C). If plasma was not utilized directly, it was stored at − 40 °C [29].

### Tick materials

*Hyalomma dromedarii* ticks were brought from camel’s market in Giza governorate. Ticks were dissected for extraction of salivary glands that were washed with 0.9% NaCl saline solution and frozen directly at − 40 °C.

### Chemicals

Thrombin (EC 3.4.21.5) from bovine plasma, fibrinogen from bovine plasma, N-(*p*-Tosyl)-Gly-Pro-Arg-*p*-nitroanilide acetate salt, ethylene glycol tetraacetic acid (EGTA), hemoglobin from bovine blood, bovine serum albumin (BSA), venom from the snake *Oxyuranus scutellatus*, and heparin-agarose were purchased from Sigma Chemical Co. Cephalit Kit for APTT, and Kit for PT were bought from bioMẻrieux. All other chemicals were of analytical grade.

### Assay of thrombin activity

**Fig. 1:**
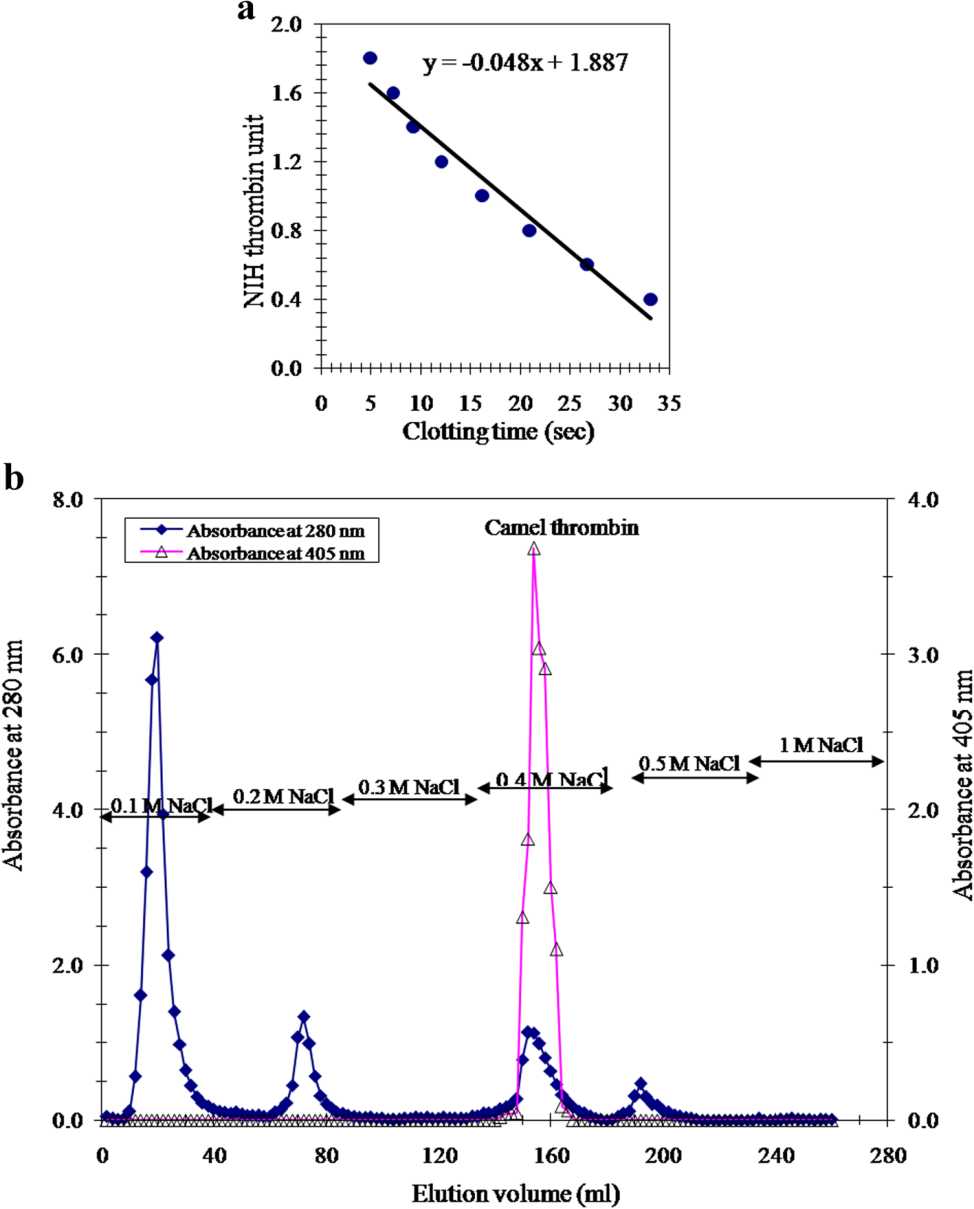
**a** Thrombin clotting time calibration curve. Clotting time is plotted against National Institute of Health (NIH) units of bovine thrombin. **b** A typical elution profile for the affinity chromatography of camel thrombin on heparin-agarose column (8 × 1.8 cm i.d.)

Chromogenic assay of thrombin was performed in a 96-well microtiter plate at 25 °C. The substrate N-(*p*-tosyl)-Gly-Pro-Arg-*p*-nitroanilide acetate salt was dissolved in dH_2_O at 1.98 mM concentration. The thrombin assay reaction mixture consists of 110 µl: 95 µl 0.05 M Tris-HCI buffer, pH 8.3 containing 0.227 M NaCI and 0.1% BSA, 5 µl enzyme solution, and 10 µl (0.18 mM) substrate*.* The reaction was started by addition of substrate, and absorbance was recorded every 5 min for 30 min at 405 nm against control lacking enzyme [30]. The clotting activity of thrombin was specified by bovine fibrinogen as substrate (5 mg/ml 0.15 M NaCl). Thrombin activity was expressed in NIH units and derived from a calibration curve for NIH bovine thrombin. The calibration curve was obtained by determination of clotting times of standard thrombin increasing concentrations (0.4–1.8 NIH unit). A total of 0.2 ml of fibrinogen solution is put onto uncoated clotting tubes at 37 °C for 30 s prior to the addition of 0.1 ml of each concentration of thrombin. The clotting times were determined and plotted against the NIH unit of thrombin. Add 0.1 ml of diluted camel thrombin preparation to an uncoated clotting tube, mix with the fibrinogen, and record clotting time. The clotting assay was done in triplicates, and the mean was taken as approximate NIH unit using the constructed calibration curve (Fig. 1a) [31].

### Purification of camel thrombin

#### Fractionation of plasma

Citrated camel plasma (1 L) was used for thrombin purification, and all successive steps were carried out at 4 °C. A total of 5 mM sodium citrate, 10 mM benzamidine-HCl, and 80 ml 1 M BaCl_2_ solution were added to 1 L of camel plasma dropwise, slowly stirred for 30 min, and then centrifuged at 3500 × *g* for 20 min for recovering the barium-adsorbed proteins. The precipitate was washed with 250 ml 0.02 M Tris–HCl buffer, pH 7.5, containing 0.15 M NaCl, 1 mM EGTA, 10 mM BaCl_2_ and 5 mM benzamidine-HCl utilizing an Omni Mixer to disperse the pellet vigorously, and the precipitate was recovered by centrifugation at 3500 × *g* for 20 min. The obtained pellet was homogenized in a Teflon-pestled homogenizer using 100 ml 0.2 M EDTA, pH 7.4, and the resulting solution was dialyzed first against 2.5 L 0.02 M Tris–HCl buffer, pH 7.5, 0.15 M NaCl, 1 mM EGTA, and 0.1 M EDTA for 5 h with rapid stirring, and dialysis was continued overnight in the same buffer without EDTA. Dialyzed sample was brought to 35% (NH4)_2_SO_4_ saturation, stirred for 20 min at 4 °C, and centrifuged at 8000 × *g* for 20 min. The filtrate was brought to 70% (NH4)_2_SO_4_ saturation, stirred for 40 min, and centrifuged at 12,000 × *g* for 30 min. The obtained pellet was dissolved in 0.02 M Tris–HCl buffer, pH 7.5 comprising 1 mM EGTA, 1 mM benzamidine-HCL, and 1 mg/ml trypsin inhibitor. This solution was dialyzed extensively with 2.5 L of this buffer and then with other 2.5 L buffer containing 0.1 M NaCl then centrifuged at 12,000 × *g* for 40 min for removing the precipitated matter [32].

#### Activation of prothrombin and heparin-agarose affinity chromatography

CaCl_2_ solution was put on the described above clarified prothrombin containing solution (10 mM final concentration) followed by addition of *Oxyuranus scutellatus* snake venom (1:40, w/w). Activation was took place at room temperature for 20 min with quiet stirring and stopped by adding stock EGTA solution to 12 mM final concentration. The final thrombin comprising solution was cooled on ice and then applied onto a heparin-agarose affinity column (8 × 1.8 cm) priorly equilibrated with 0.02 M Tris–HCl buffer pH 7.5, 0.1 M NaCl, and 1 mM EGTA. After sample loading, the column was subjected to an exhaustive washing with the same buffer for removing the nonbound proteins. Bound proteins were eluted with stepwise gradient (0.1–1 M NaCl) in the same buffer and collection of 1 ml fractions with a flow rate of 20 ml/h.

#### Electrophoretic analysis

Seven percent native polyacrylamide gel electrophoresis was carried out [33]. Twelve percent SDS-PAGE was performed for determination of subunit molecular weights [34, 35]. Molecular weight markers, phosphorylase b (94 kDa), bovine serum albumin (67 kDa), ovalbumin (43 kDa), carbonic anhydrase (30 kDa), soybean trypsin inhibitor (20.1 kDa), and lactalbumin (14.4 kDa) were used for calibration. Coomassie Brilliant Blue R-250 was used for staining the proteins.

#### Protein determination

Proteins were estimated through the dye binding assay method by using bovine serum albumin as a standard protein [36].

## Results

### Thrombin purification from camel plasma

The activated prothrombin-enriched fraction with snake venom (*Oxyuranus scutellatus*) was applied directly to heparin-agarose column for affinity chromatography. A typical elution profile (Fig. 1b) showed one thrombin activity peak was eluted with 0.4 M NaCl as detected by the thrombin chromogenic assay and represented by the absorbance at 405 nm. The purification procedure of camel thrombin is summarized in Table 1. The specific activity of the purified camel thrombin was 3242 NIH thrombin unit/mg protein with 10.35-fold and 88.5% recovery.

**Table 1:** Purification scheme of the camel thrombin

Purification step	Total protein (mg)	Total activity (units)	Specific activity	Yield (%)	Purification fold
35–70% (NH4)_2_SO_4_ activated fraction	120	37,591	313.26	100	1
Heparin-agarose fraction	10.3	33,290	3242	88.5	10.35

### Electrophoretic analysis of camel thrombin

**Fig. 2:**
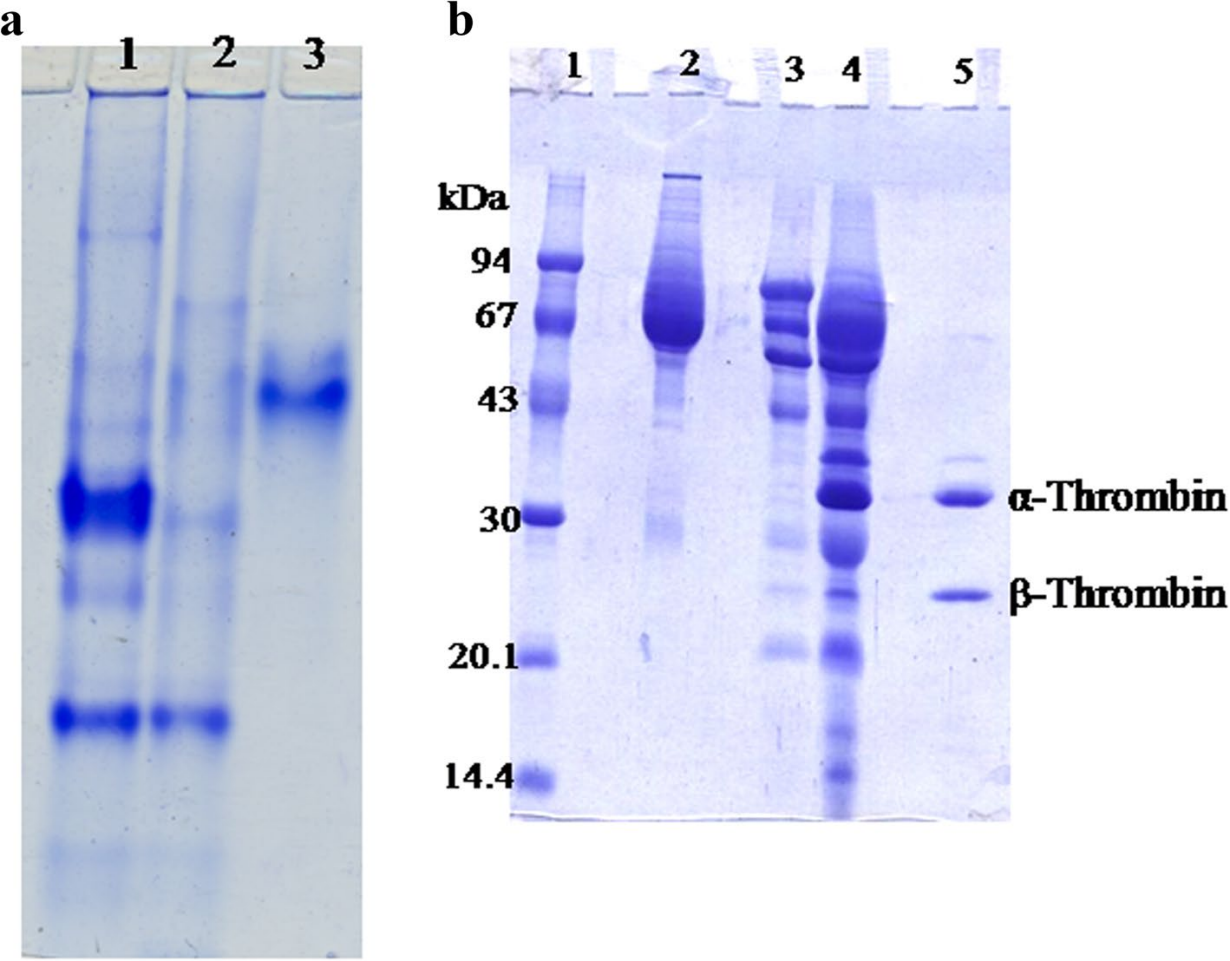
**a** Native 7% PAGE for the purification steps of camel thrombin: (1) the prothrombin-containing sample, (2) the activated prothrombin-containing sample, and (3) the purified camel thrombin. **b** 12% SDS PAGE: (1) molecular weight markers, (2) plasma, (3) 35–70% saturated (NH_4_)_2_SO_4_ fraction enriched with prothrombin, (4) activated prothrombin into thrombin by snake venom, (5) the purified camel thrombin

The purified camel thrombin eluted from heparin-agarose column was analyzed by 7% native PAGE (Fig. 2a) that turned out to be homogenous as shown by one protein band. Purified camel thrombin was also analyzed by 12% SDS PAGE that showed one major protein band of 37 kDa and a minor smaller band of 28 kDa. The camel prothrombin molecular weight was appeared as 72 kDa protein band (Fig. 2b).

### Effect of substrate concentration and pH on the camel thrombin activity

**Fig. 3:**
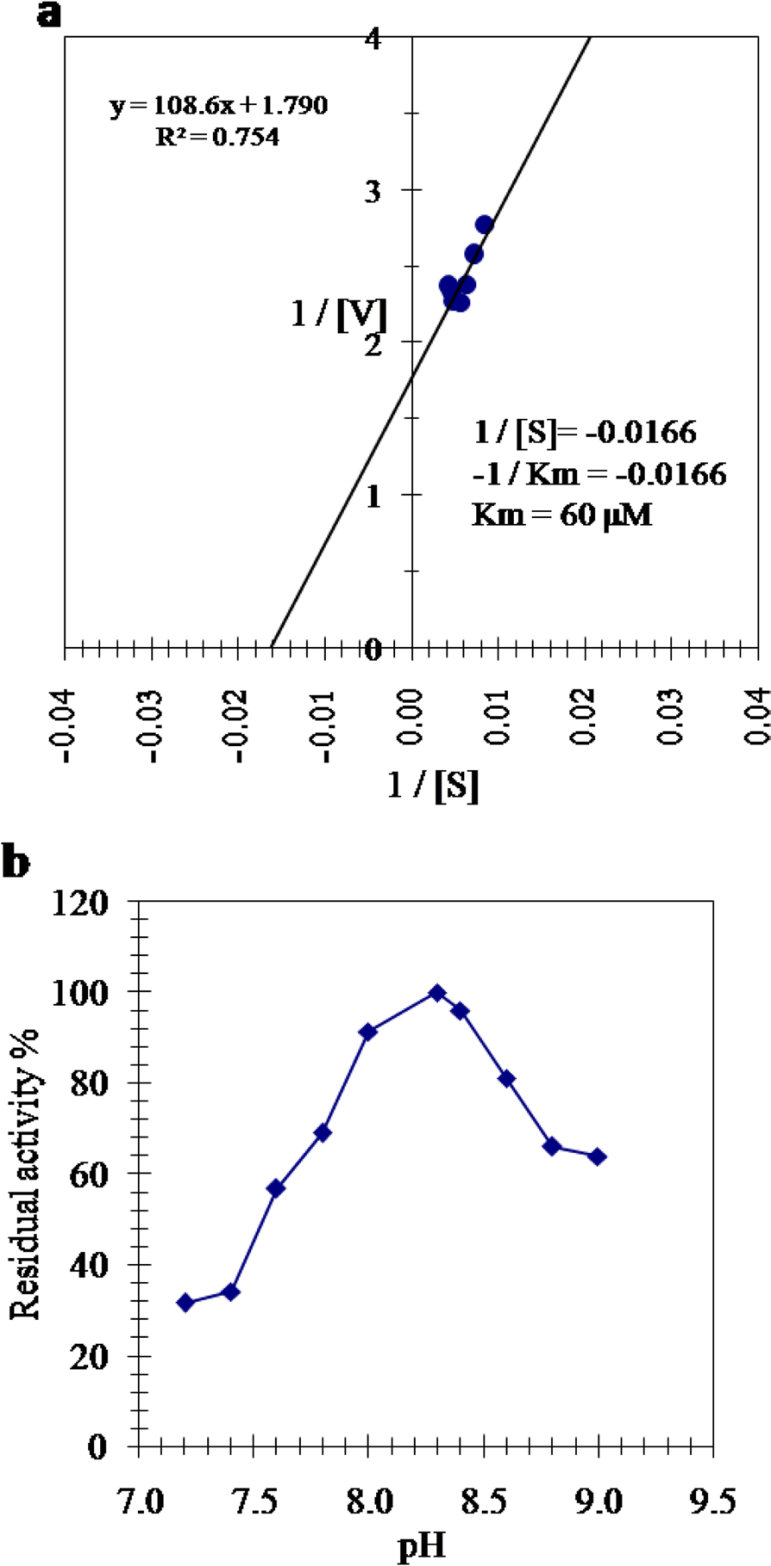
**a** Lineweaver–Burk plot relating the reciprocal of the reaction velocity of the purified camel thrombin to the reciprocal of the substrate concentration in μΜ (1/[S]) by using N-p-Tosyl-Gly-Pro-Arg-p-nitroanilide acetate as substrate. The reaction velocity is the change in absorbance at 405 nm per 30 min. **b** Effect of pH on the camel thrombin activity using N-p-Tosyl-Gly-Pro-Arg-p-nitroanilide acetate as a substrate in 0.05 M Tris–HCl buffer of various pH values

Lineweaver–Burk plot for reciprocal of the camel thrombin reaction velocity (1/v) and substrate concentration (1/[S]) was constructed yielding a *Km* value of 60 µM N-(*p*-Tosyl)-Gly-Pro-Arg-*p*-nitroanilide acetate (Fig. 3a). Effect of pH on the activity of camel thrombin was inspected using 0.05 M Tris–HCl buffer, pH (7.2–9.0), and N-(*p*-Tosyl)-Gly-Pro-Arg-*p*-nitroanilide acetate as substrate. The thrombin from camel plasma demonstrated its optimum activity at pH 8.3 (Fig. 3b).

### Effect of various inhibitors on the camel thrombin activity

Effect of various inhibitors on the activity of camel thrombin was carried out (Table 2). The benzamidine HCl, cysteine, EDTA, EGTA, *β*-mercaptoethanol, and trypsin inhibitor showed slight inhibition, while PMSF had a strong influence on the purified camel thrombin. A total of 2.5 μM camel tick salivary gland thrombin inhibitor previously purified [20] inhibited 97% of the purified camel thrombin activity.

**Table 2:** Effect of different inhibitors on the purified camel thrombin

Inhibitor	Concentration	Camel thrombin inhibition %
No inhibitor	–-	0.0
Benzamidine HCl	5 mM	13.5
Cysteine	5 mM	5.5
EDTA	5 mM	11.0
EGTA	5 mM	14.5
β-Mercaptoethanol	5 mM	35.0
PMSF	2 mM	93.0
Soybean trypsin inhibitor	5 mM	38.7
Camel tick salivary gland thrombin inhibitor	2.5 µM	97.0

^*^These values represent % of the control and the means of triplicate experiments

### Kinetics of camel thrombin inhibition with camel tick salivary gland thrombin inhibitor

**Fig. 4:**
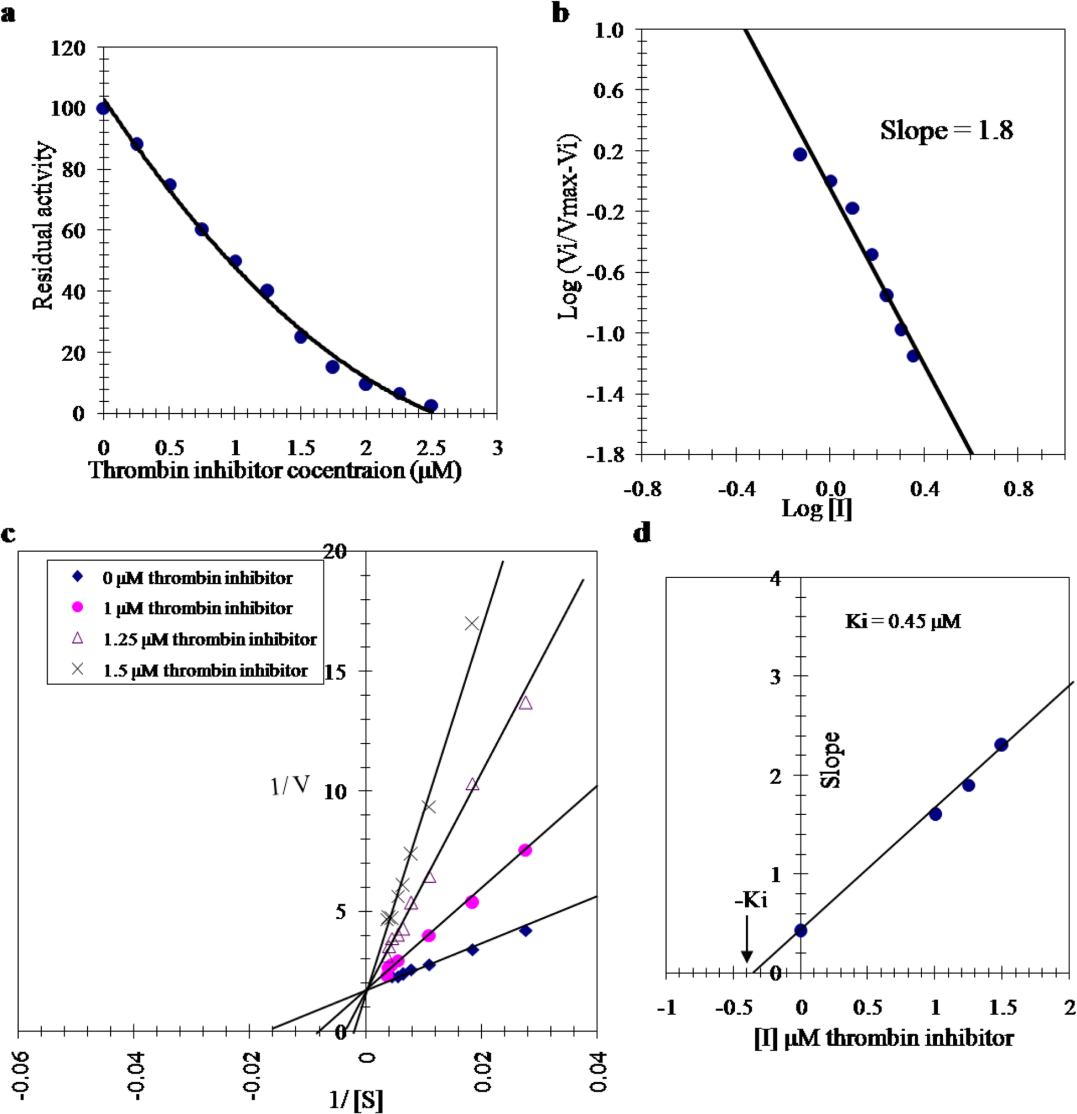
**a** The effect of varying concentrations of the camel tick salivary gland thrombin inhibitor on the activity of camel thrombin. **b** Hill plot for the inhibition of the activity of camel thrombin by increasing concentrations of the camel tick salivary gland thrombin inhibitor. **c** Lineweaver–Burk plots showing the type of inhibition of the camel thrombin by the camel tick salivary gland thrombin inhibitor. The activity of a constant amount of camel thrombin was measured with varying concentrations of the substrate N-p-Tosyl-Gly-Pro-Arg-p-nitroanilide acetate in the absence and presence of three various concentrations of salivary gland thrombin inhibitor. **d** Determination of the inhibition constant (Ki) value for inhibition of camel thrombin activity by the camel tick salivary gland thrombin inhibitor. The plotted slope values were determined from the lines of reciprocal plots of the different inhibitor concentrations

Effect of different concentrations (0–3.75 μM) of camel tick salivary gland thrombin inhibitor on the activity of camel thrombin was carried out. A total of 97% maximum inhibition of camel thrombin was achieved by 2.5 μM salivary gland inhibitor (Fig. 4a). On constructing the Hill plot when values of log (Vi/Vmax–Vi) were drew against log [I] of the thrombin inhibitor, a direct line was acquired with a slope of 1.8 (Fig. 4b). Lineweaver–Burk plot indicated that the inhibition of camel thrombin with the salivary gland inhibitor is competitive (Fig. 4c) with a *Ki* value 0.45 μM (Fig. 4d).

## Discussion

The main role of thrombin is to catalyze the transformation of fibrinogen to fibrin necessary for thrombus generation. Thrombin activates factor XIII to cross with fibrin for stabilizing, promoting, and amplifying the clot formation via activation of other clotting factors [7, 37]. Thrombin also plays a key role in other physiological processes as it has been used clinically as a hemostatic agent to stanch oozing hemorrhages in the field of surgery and effective in stopping bleeding on local wounds and in the gastrointestinal organs [38, 39]. It is also utilized as a constituent in surgical binding factors in human surgeries and in the meat industry [10, 12].

In the present study, camel prothrombin was efficaciously fractionated from other coagulation agents via utilizing barium citrate adsorption. The dialysis of the barium-adsorbed plasma proteins mixture in buffer containing EDTA led to the releasing of proteins from this adsorbed complex, and this is the most critical step in the recovery of prothrombin from plasma. The camel thrombin was prepared with a single affinity chromatographic step on heparin-agarose column (Fig. 1b) with a specific activity of 3242 NIH units/mg proteins, 10.35-fold, and 88.5% recovery (Table 1). The prepared camel thrombin was found to be homogenous as proved by the 7% native PAGE (Fig. 2a). Thrombin was purified from human plasma with a specific activity of 4000 NIH units/mg protein [32] and 2400 IU/mg protein [40], from bovine plasma 4018 NIH units/mg [31], and from salmon blood 1000 units/mg protein [10].

In this study, the purified camel thrombin contained one major band with apparent molecular weight of 37 kDa in consistence with the size prospected for *α*-thrombin and a minor smaller protein of 28 kDa in consistence with the size prospected for *β*-thrombin. Molecular weight of camel prothrombin was approximately appeared as 72 kDa (Fig. 2a). On comparison of the reported thrombin molecular weights, the molecular weight of bovine prothrombin was 72 kDa, the activation of which resulted in two single-chain molecules of 39 kDA and 24 kDa [41]. SDS-PAGE showed the bovine thrombin as one major band of 35 kDa and a minor protein suggested being *β*-thrombin [31]. The human prothrombin and thrombin were reported to be 70 and 34.8 kDa [32] and 72 kDa and 37 kDa [39]. Three forms of human thrombin were visualized as follows: 37 kDa *α*-thrombin, 27 kDa *β*-thrombin, and 13 kDa *γ*-thrombin [40]. The salmon thrombin molecular weight was 37 kDa [10].

In this study, the *Km* value of the purified camel thrombin was 60 µM of N-(*p*-Tosyl)-Gly-Pro-Arg-*p*-nitroanilide acetate (Fig. 3a) indicating the high affinity of the purified camel thrombin toward N-*p*-Tosyl-Gly-Pro-Arg-*p*-nitroanilide acetate. The purified camel thrombin exhibited its maximum activity at pH 8.3 (Fig. 3b). In consistent with this result, salmon thrombin exhibited its maximum activity at pH 8.5, while human thrombin exhibited its maximum activity at pH 8.0 [10]. The purified camel thrombin was partially inhibited with benzamidine HCl and EGTA. It was slightly inhibited with cysteine and EDTA which indicated that it is neither cysteinyl proteinase nor a metalloenzyme. Inhibition of camel thrombin by *β*-mercaptoethanol denotes the existence of a disulfide bond in the thrombin molecule and trypsin inhibitor denoting it as a serine protease. The serine protease inhibitor PMSF was found to be the most powerful inhibitor of camel thrombin indicating that the enzyme active site contains a serine residue (Table 2). The titration curve of the salivary gland thrombin inhibitor toward the camel thrombin activity (Fig. 4a) emphasized inhibition of camel thrombin with the maximum inhibition (97%) was reached by 2.5 μM of the inhibitor, while 3.75 μM of this inhibitor inhibited 82% of bovine thrombin [20]. Two binding sites are derived for the salivary gland inhibitor on camel thrombin because the slope of the Hill plot was found as 1.8 (Fig. 4b). Existence of the salivary gland thrombin inhibitor did not alter the value of *Vmax* and increases the *Km* value denoting a competitive inhibition of camel thrombin by camel tick salivary gland thrombin inhibitor (Fig. 4c) with *Ki* value of 0.45 μM (Fig. 4d). The salivary gland thrombin inhibitor had only one binding site on bovine thrombin and *Ki* value of 0.55 μM [20] confirming the higher susceptibility of the camel thrombin as the proper and natural target enzyme to the inhibitor than the bovine thrombin. A competitive inhibition of thrombin was achieved by americanin, savignin and NTI-2 [42–44], and a noncompetitive inhibition by NTI-1 [42]. Ki values of dipetarudin, a chimeric thrombin inhibitor from the assassin bug *Dipetalogaster maximus*, have been reported to be 446 fM [45] and 399 ± 83 fM of dipetarudin fron *Pichia pastoros* [46].

## Conclusion

In conclusion, this study provides simple, reproducible, and appropriate method for purification of thrombin from camel plasma which is very valuable as hemostatic factor. The purified camel thrombin was found more susceptible toward the camel tick salivary gland thrombin inhibitor than bovine thrombin. This thrombin inhibitor can be applicable in two major directions: (1) in tick control, since antibodies directed to this inhibitor may block the successful feeding of ticks, and (2) therapeutic purposes where the potency and specificity of this thrombin inhibitor indicate that it may be effective in the treatment of thrombosis.

## Data Availability

All data generated or analyzed during this study are included in this published article and available upon request from the corresponding author.

## Abbreviations

PMSF: Phenylmethylsulfonyl fluoride
BSA: Bovine serum albumin
PAGE: Polyacrylamide gel electrophoresis
EGTA: Ethylene glycol tetraacetic acid
